# High serum urokinase-type plasminogen activator receptor correlates with sepsis-induced cardiomyopathy in sepsis patients

**DOI:** 10.3389/fmed.2026.1789588

**Published:** 2026-04-09

**Authors:** Jianzhuo He, Tiesheng Wu, Guangping Wu, Yuxin Huang, Xin Yin, Minzhou Zhang, Bangjiang Fang, Liheng Guo

**Affiliations:** 1 The Second Affiliated Hospital of Guangzhou University of Chinese Medicine, Guangzhou, China; 2 Post-Doctoral Workstation of Shanghai University of Traditional Chinese Medicine, Shanghai, China; 3 The Second Clinical College of Guangzhou University of Chinese Medicine, Guangzhou, China; 4 Department of Emergency, Longhua Hospital, Shanghai University of Traditional Chinese Medicine, Shanghai, China

**Keywords:** sepsis-induced cardiomyopathy, uPAR, antibody microarray, diagnostic biomarker, myocardial dysfunction

## Abstract

**Background:**

Sepsis-induced cardiomyopathy (SIC), a major complication of sepsis characterized by myocardial injury and cardiac dysfunction, significantly increases mortality. Inflammation plays a central role in SIC, yet specific biomarkers for early diagnosis remain limited. This study aimed to identify inflammatory cytokines associated with SIC and evaluate their diagnostic potential.

**Methods:**

Serum levels of 40 immune-related cytokines were measured in 25 SIC patients, 25 sepsis patients, and 10 healthy controls using antibody microarrays. Correlation analysis examined associations between cytokines and clinical parameters, while Receiver operating characteristic (ROC) curves and logistic regression assessed their predictive accuracy for myocardial dysfunction.

**Results:**

Urokinase-type plasminogen activator receptor (uPAR) was found to be significantly elevated in SIC patients compared to sepsis patients and healthy controls. Furthermore, uPAR levels were found to significantly and positively correlated with Acute Physiology and Chronic Health Evaluation II (APACHE II) score, sequential organ failure assessment score (SOFA), B-type natriuretic peptide (BNP), as well as the use and duration of vasopressor therapy, and negatively correlated with fraction of shortening (FS). ROC curve analysis showed that the predictive value of uPAR was inferior to that of BNP, cardiac troponin I (cTnI), and FS; while superior to that of left ventricular end-diastolic dimension (LVEDD). Logistic regression analysis showed that uPAR significantly affected the occurrence of myocardial dysfunction.

**Conclusion:**

Our study suggests that uPAR may serve as a candidate biomarker associated with SIC. Combining uPAR with other markers could enhance diagnostic accuracy. Additionally, uPAR levels may be associated with vasopressor use in SIC patients.

## 1 Introduction

Sepsis is a potentially life-threatening condition caused by a dysregulated host response to infection, resulting in multiple organ failure, shock, disability, and even death. Sepsis leads to morbidity and mortality in intensive care units (ICU) (1). Sepsis-induced cardiomyopathy (SIC), also known as sepsis-induced myocardial dysfunction (SIMD) or sepsis myocardial injury, is one of the most serious complications and major causes of death in sepsis. Additionally, it directly affects the prognosis of sepsis. Myocardial dysfunction is a consequence of severe sepsis, and approximately 50% of sepsis patients exhibit signs of myocardial dysfunction (2). Myocardial injury is the core pathogenesis of SIC, while cardiac dysfunction is a clinical manifestation with complex pathogenesis. Currently, there is no specific treatment for this condition. SIMD is characterized by biventricular dysfunction, decreased ejection fraction (EF), and ventricular dilation. SIMD can result in an inadequate cardiac output response to decreased afterload, resulting in significantly higher mortality in patients with sepsis (3).

Left ventricular ejection fraction (LVEF) is most commonly used to diagnose myocardial dysfunction on echocardiography. However, echocardiography requires a professional technician, and lacks continuous monitoring. Elevated cardiac troponin T (cTnT), cardiac troponin I (cTnI), and B-type natriuretic peptide (BNP) levels have been identified in septic patients with cardiac dysfunction, and correlated with adverse outcomes in sepsis (4, 5). Additionally, systemic inflammatory response syndrome (SIRS) is aggravated by inappropriate immune response, which results in myocardium being functionally injured by inflammatory cytokines. Previous studies have indicated that tumor necrosis factor (TNF-α) is a circulating myocardium-depressing factor, the expression of which could decrease myocardial contractility (6), and inhibit the expressions of cardiac ICAM-1 and VCAM-1 ameliorated myocardial injury in both lipopolysaccharide (LPS) and cecal ligation and puncture (CLP) models of sepsis (7). Cytokines can activate endothelial cells, cardiac fibroblasts, and cardiomyocytes, which further stimulate inducible nitric oxide synthase (iNOS) expression and result in myocardial dysfunction in sepsis (8). Therefore, it may be useful to measure circulating inflammatory cytokines in sepsis patients with myocardial dysfunction to explore novel biomarkers for early diagnosis of SIC.

Antibody microarrays are a novel technology that rapidly and simultaneously detect multiple proteins in a high-throughput format. In the present study, an Immune Response Antibody Array was used to detect 40 human immune response-associated cytokines to improve the early diagnosis of SIC.

## 2 Materials and methods

### 2.1 Study design

The study was a single-center, prospective, observational cohort study that was conducted at Guangdong Provincial Hospital of Traditional Chinese.

### 2.2 Study population and blood sample collection

Fifty patients admitted to the ICU, who fulfilled the criteria for sepsis and septic shock (9) were enrolled for the study between March 2017 and January 2018. Exclusion criteria were: age < 18 years, history of myocardial infarction, angina pectoris, acute coronary syndromes, renal dysfunction, liver insufficiency, HIV infection, and chronic heart failure. The study participants were divided into sepsis-induced cardiomyopathy group (SIC group, *n* = 25) and sepsis without myocardial dysfunction group (sepsis group, *n* = 25), based on ultrasound cardiogram (UCG) and cardiac markers (10). The factors taken into consideration were: diagnosis of sepsis and elevation of myocardial markers (cTnI or BNP, >99th percentile of normal); systolic dysfunction: echocardiographic findings of LVEF <50%; diastolic dysfunction: LVEF>50% and left ventricular end-diastolic dimension (LVEDD) < 50 mm, Doppler ultrasound: E/A ratio (≤1) of mitral orifice blood flow between rapid filling and atrial systolic period, BNP > 200 pg./mL, left ventricular diameter shortening rate (FS) > 25% with normal or thickened wall thickness, yet slowed down left ventricular filling rate.

Patients with sepsis were evaluated for SIC within 12 h of sepsis confirmation/ICU admission by echocardiography and cardiac biomarker assessment. Following SIC classification and enrollment into the study groups, blood samples for suPAR measurement were obtained within 24 h.

### 2.3 Antibody array assay

A human antibody array (RayBio Quantibody^®^ Human Immune Response Array, Cat#: QAH-IMR-1, RayBiotech, Norcross, GA, USA) was used to quantify and simultaneously detect 40 immune response-associated cytokines. Briefly, after incubation with blocking buffer, arrays preprinted with 40 primary antibodies were incubated with a standard mix at gradient concentrations and serum samples, which were diluted two folds overnight at 4 °C. After washing, biotin-conjugated detection antibody mix was added to the array pools and incubated for 2 h at room temperature. Subsequently, Cy3-conjugated streptavidin was added to the array pools for binding with biotin-conjugated detection antibodies, and further incubated for 2 h at room temperature. Finally, the slides were scanned with an InnoScan 300 Microarray Scanner (Innopsys, France) and the signal values were read using Mapix software. The concentrations of 40 immune response-associated cytokines in serum samples were calculated using corresponding standard curves drawn by RayBiotech QAH-IMR-1 analysis tool. This experiment was performed once, and each analyte on the array was printed in quadruplicate. The intra-assay CV was <10%.

Blood samples were collected in clean and dry vacuum tubes without additives, and serum was separated by centrifugation at 2,000 rpm for 10 min at 4 °C. The serum samples were aliquoted immediately and stored at −80 °C until antibody array analysis. Prior to analysis, samples were thawed appropriately, and repeated freeze–thaw cycles were avoided as much as possible.

### 2.4 Statistical analysis

Comparisons between groups were performed by one-way analysis of variance (ANOVA), followed by multiple comparisons with *post-hoc* Bonferroni test using SPSS software (version 22.0; SPSS Inc., Chicago, IL, USA). *p-*values< 0.05 were considered statistically significant. All data were presented as mean ± standard deviation (SD). Fold change (FC) was calculated to determine the relative expression levels of cytokines. Correlation analysis was performed using GraphPad Prism 5 software, and logistic regression was performed using SPSS software (version 22.0; SPSS Inc., Chicago, IL, USA). Venn Diagram analysis was performed using the website.^1^

## 3 Results

### 3.1 Patient characteristics

The UCG parameters (LVEF, FS, LVEDD) and cardiac markers (cTnI BNP) were significantly different between the SIC and sepsis groups. Furthermore, the Acute Physiology and Chronic Health Evaluation (APACHE) II, Sequential Organ Failure Assessment (SOFA) scores, and number of patients using vasopressors in the SIC group were significantly higher compared to the sepsis group. No significant differences were observed in age or sex among the SIC, sepsis, and healthy groups. Patient characteristics are presented in Table 1.

**Table 1:** Baseline characteristics of trial participants.

Variable	SIC group (*n* = 25)	Sepsis group (*n* = 25)	Healthy control group (*n* = 10)	*p-*value
Age (years)	69.3 ± 12.5	71.4 ± 11.7	66.5 ± 8.15	0.33
Male	18 (72%)	16 (64%)	7 (70%)	0.83
Site of infection				
Lung	22 (88%)	18 (72%)	0	0.16
Bloodstream	4 (16%)	3 (12%)	0	0.69
Intra-abdominal	9 (36%)	3 (12%)	0	0.05
Urinary tract	1 (4%)	4 (16%)	0	0.16
Central nervous system	1 (4%)	0	0	0.32
Other source^*^	0	2 (8%)	0	0.15
Vasopressor	15 (60%)	6 (24%)	0	0.01^*^
Mechanical ventilation	24 (96%)	20 (80%)	0	0.09
CRRT	3 (12%)	3 (12%)	0	1.00
APACHE II	21.0 ± 6.5	14.4 ± 9.2	5.2 ± 1.4	0.01^*^
SOFA	13.2 ± 4.7	5.9 ± 4.8	0.5 ± 1.0	0.00^*^
HR (bpm)	111.5 ± 25.1	103.8 ± 23.4	76.5 ± 14.8	0.268
MAP (mmHg)	87.5 ± 21.3	93.9 ± 15.7	96.8 ± 15.6	0.229
UCG parameters				
LVEF (%)	45.4 ± 13.6	66.3 ± 4.3	65.6 ± 6.7	0.00^*^
FS (%)	24.4 ± 8.6	37.2 ± 6.7	36.2 ± 5.0	0.00^*^
LVEDD (mm)	49.1 ± 9.5	42.0 ± 8.1	45.7 ± 2.3	0.01^*^
E/A (≤1)	21 (84%)	20 (80%)	8 (80%)	
Laboratory results				
Alb (g/L)	35.2 ± 5.2	32.4 ± 6.2	41.8 ± 3.8	0.09
Glu (mmol/L)	8.4 ± 3.0	11.1 ± 7.0	7.4 ± 3.0	0.08
Scr (umol/L)	178.7 ± 160.0	113.2 ± 134.3	102.2 ± 54.8	0.12
cTnI (ng/ML)	0.44 ± 0.86	0.04 ± 0.06	0.01 ± 0.01	0.027^*^
LAC (mmol/L)	4.0 ± 3.2	3.1 ± 5.1	/	0.48
Procalcitonin (ug/L)	24.4 ± 35.3	9.4 ± 18.8	/	0.07
C-reactive protein (mg/L)	110.0 ± 116.0	123.0 ± 130.1	4.3 ± 6.3	0.71
BNP (pg/mL)	1313.9 ± 1469.9	187.2 ± 175.3	/	0.005^*^
The duration of vasopressor	3.3 ± 6.5	1.6 ± 3.6	0	0.023^*^
28-day mortality rate	3 (12%)	4 (16%)	0	0.69

Data are presented as mean ± standard deviation, median (interquartile range), or *n* (%) unless indicated otherwise.

^*^Other sources of infection were soft-tissue infection (*n* = 5), mediastinitis (*n* = 2), pleural empyema (*n* = 3), septic arthritis (*n* = 3), and unknown origin (*n* = 14).

CRRT, continuous renal replacement therapy; APACHE II, Acute Physiology and Chronic Health Evaluation II score; SOFA, Sequential Organ Failure Assessment score; HR, heart rate; MAP, mean arterial pressure; UCG, Ultrasound Cardiogram; LVEF, left ventricular ejection fraction; FS, Fraction of shortening; LVEDD, left ventricular end-diastolic dimension; E/A (≤1), E/A ratio; Alb, albumin; Glu, glucose; Scr, serum creatinine concentration; cTnI, cardiac troponin I; LAC, Blood Lactic Acid; BNP, Brain Natriuretic Peptide.

### 3.2 Identification of uPAR as a SIC-specific cytokine by antibody array analysis

To identify cytokines specifically associated with SIC, serum levels of 40 immune response-associated cytokines were measured across the SIC, sepsis, and healthy control groups.

The filtering strategy proceeded in two steps: first, statistical analysis identified proteins that differed significantly among the three groups; second, Venn diagram analysis was applied to identify SIC-specific cytokines, defined as cytokines with differential levels in the SIC group compared to both sepsis and healthy groups, but without differential levels between the sepsis and healthy groups—thereby excluding general sepsis-related inflammatory signals.

Statistical analysis revealed that four proteins (resistin, thrombomodulin, uPAR, and CD163) differed between the SIC and sepsis groups; six proteins (lipocalin-2, CRP, CD14, IL-13, uPAR, and ICAM-1) differed between the SIC and healthy groups; and seven proteins (lipocalin-2, CRP, CD14, IL-13, PF4, FAS L, and ICAM-1) differed between the sepsis and healthy groups (Table 2).

**Table 2:** The antibody array data of differential proteins.

Protein	SIC	Sepsis	Healthy	SIC vs. sepsis		SIC vs. healthy		sepsis vs. healthy	
	Mean ± SD	Mean ± SD	Mean ± SD	*p*-value	Fold change	*p-*value	Fold change	*p*-value	Fold change
CD14	10019.3 ± 3182.9	8632.9 ± 2619.8	15638.5 ± 7610.4	0.689	1.161	0.001	0.641	0.000	0.552
CD163	129468.4 ± 98032.9	71333.1 ± 64436.6	88083.0 ± 82704.3	0.048	1.815	0.563	1.470	1.000	0.810
CRP	31230.2 ± 8843.3	32582.3 ± 7675.7	49861.7 ± 19637.4	1.000	0.959	0.000	0.626	0.000	0.653
FAS L	65.2 ± 67.6	46.8 ± 35.6	108.8 ± 31.3	0.623	1.394	0.080	0.599	0.006	0.430
ICAM-1	31292.8 ± 14579.0	28579.7 ± 12387.7	45180.1 ± 18179.6	1.000	1.095	0.037	0.693	0.009	0.633
IL-13	3.8 ± 3.5	3.0 ± 2.4	6.8 ± 2.2	0.960	1.275	0.024	0.561	0.003	0.440
Lipocalin-2	619.5 ± 143.7	637.6 ± 174.2	1059.3 ± 201.4	1.000	0.972	0.000	0.585	0.000	0.602
PF4	3030.5 ± 1151.6	2346.5 ± 864.9	3687.2 ± 1081.3	0.067	1.291	0.280	0.822	0.003	0.636
Resistin	8018.8 ± 8833.0	1897.2 ± 937.6	3083.8 ± 1663.5	0.001	4.227	0.080	2.600	1.000	0.615
Thrombomodulin	2145.9 ± 1503.6	1188.7 ± 701.7	1739.1 ± 670.2	0.010	1.805	0.993	1.234	0.570	0.684
uPAR	31051.6 ± 25864.0	15672.1 ± 14484.7	11542.4 ± 4955.0	0.020	1.981	0.028	2.690	1.000	1.358

**Figure 1:**
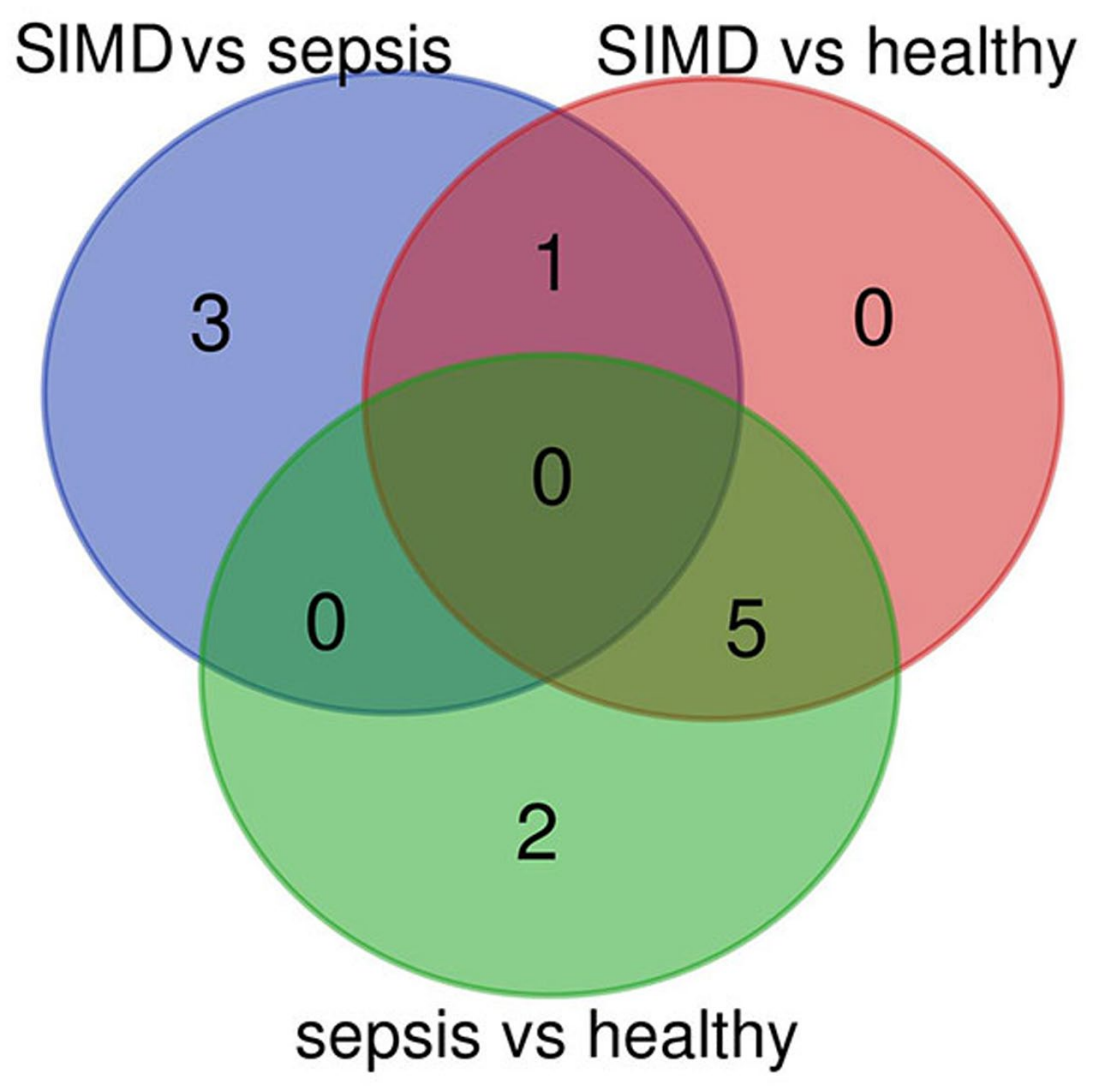
Venn diagram analysis. The differential proteins between any two groups were used to search specific SIC cytokines which are defined as the cytokines with differential levels in SIC group compared with sepsis and healthy groups, but without differential levels between sepsis and healthy groups. Venn diagram analysis showed there was a specific SIC cytokine (SIC, *n* = 25; sepsis, *n* = 25; healthy, *n* = 10).

**Figure 2:**
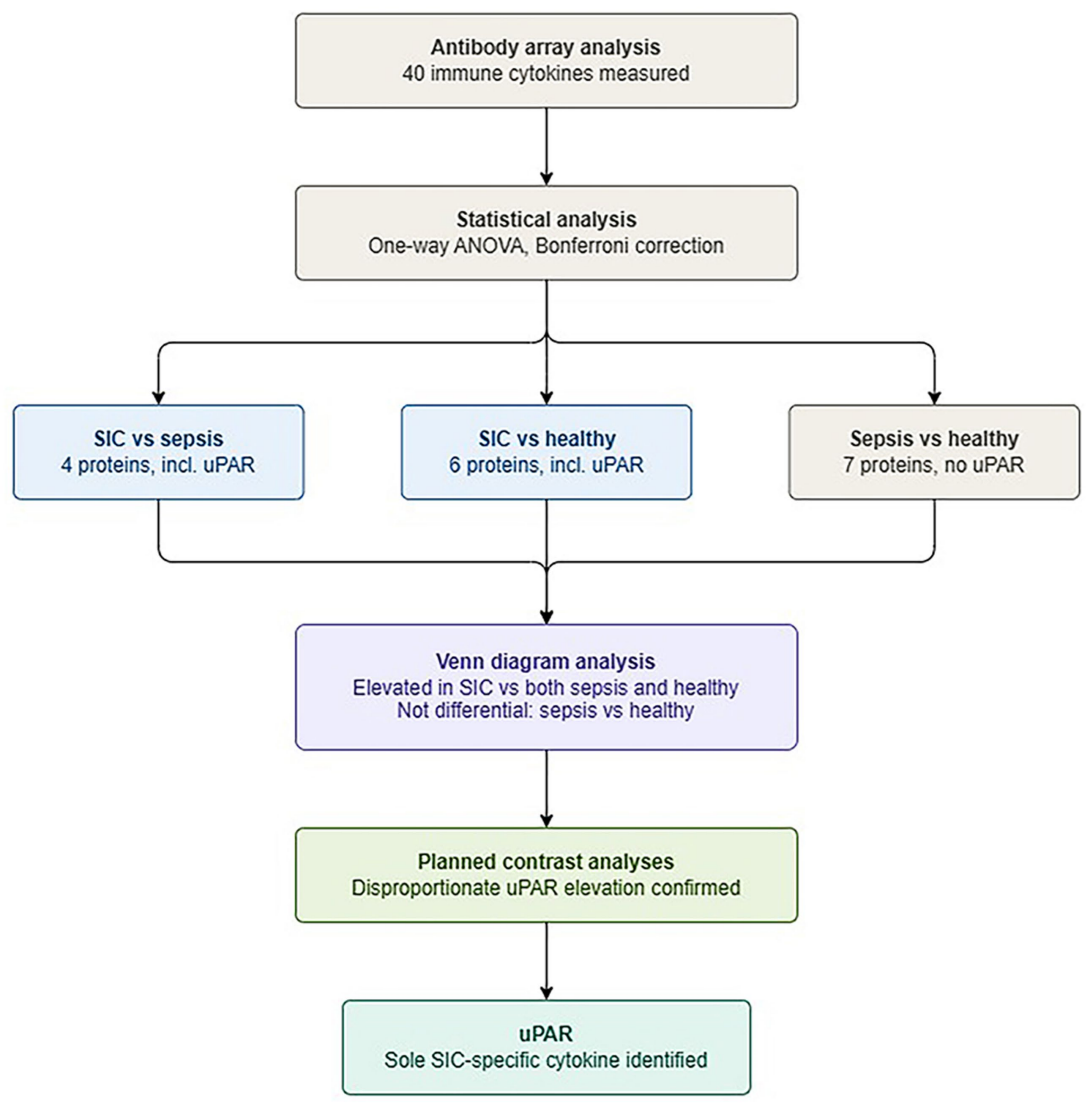
Filtering process for identification of uPAR as a SIC-specific cytokine.

**Figure 3:**
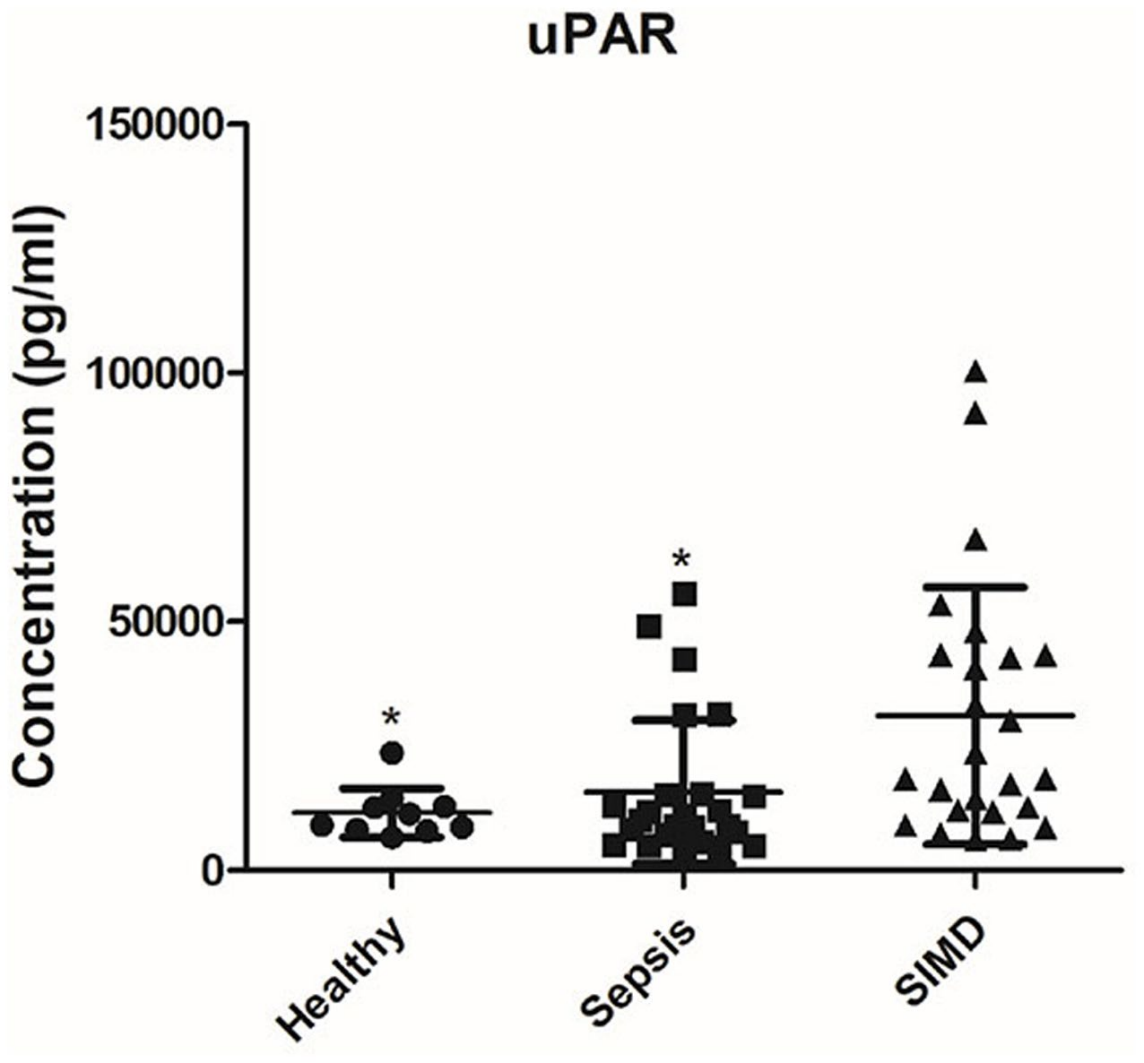
UPAR levels among three groups. The relative expression levels of uPAR in antibody array detection were showed by scatter diagram. Each point represents one individual patient sample. Group comparisons were performed by one-way ANOVA followed by Bonferroni *post-hoc* test. *p < 0.05 versus SIC group (SIC, *n* = 25; sepsis, *n* = 25; healthy, *n* = 10).

**Figure 4:**
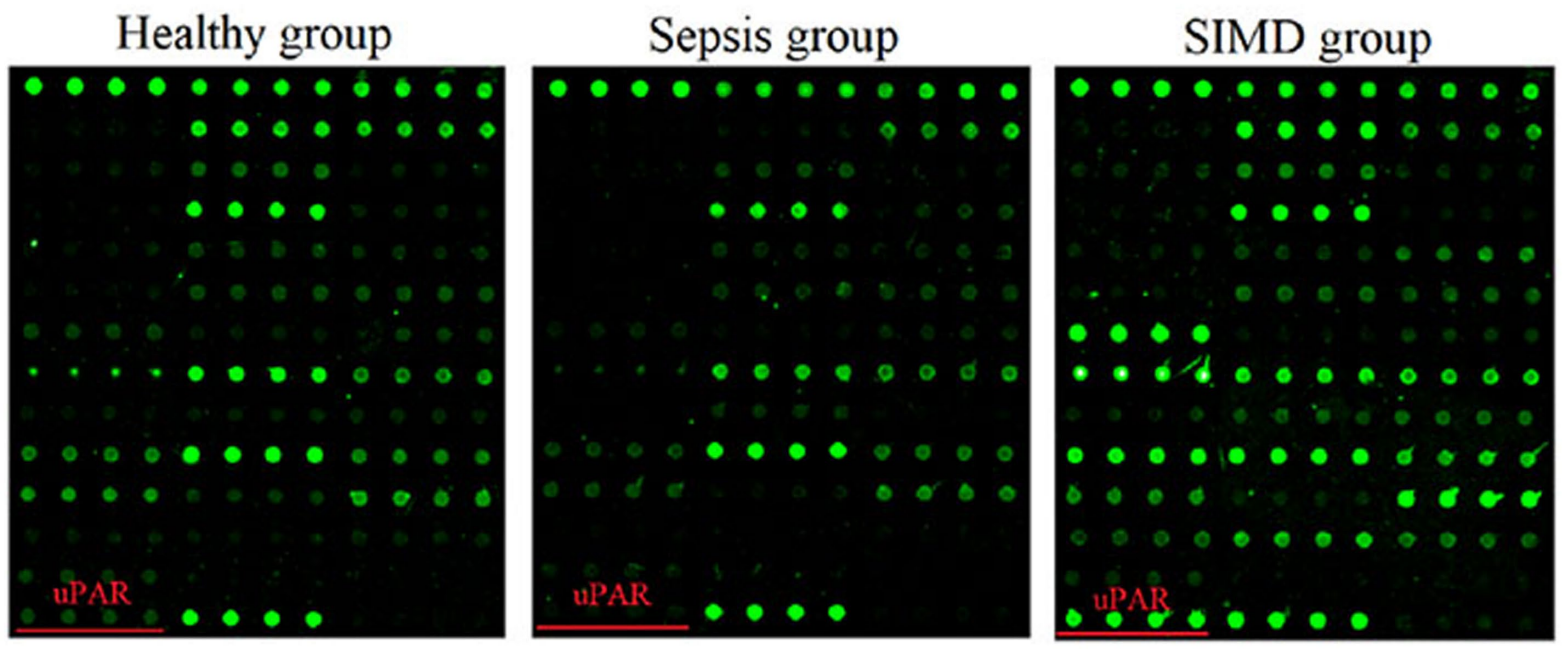
The antibody array profiles. The location of uPAR is labeled with red line. The levels of proteins are proportional to their fluorescence intensities in antibody array profiles. In these arrays, the antibody of each protein was printed in four duplicates (SIC, *n* = 25; sepsis, *n* = 25); healthy, *n* = 10).

Applying the SIC-specificity criterion described above, Venn diagram analysis identified uPAR as the sole cytokine meeting all criteria (Figure 1). To further confirm this finding beyond statistical threshold dependency, planned contrast analyses were conducted. The results showed that uPAR levels were significantly higher in SIC patients compared to sepsis patients (contrast value = 15,380, *t* = 2.812, *p* = 0.007) and healthy controls (contrast value = 19,509, *t* = 2.697, *p* = 0.009), whereas the difference between sepsis patients and healthy controls was substantially smaller in magnitude (contrast value = 4,130) and did not reach statistical significance (*p* = 0.570). These results indicate that uPAR elevation in SIC represents a disproportionate increase beyond the general sepsis-associated inflammatory response. The array profiles and histogram further confirmed higher levels of uPAR in the SIC group (Figures 2-4).

### 3.3 Correlation of uPAR levels with clinical parameters

Correlation analysis showed that uPAR levels significantly positively correlated with APACHE II, SOFA, BNP, along with the use and duration of vasopressor therapy, and negatively correlated with FS (Pearson r_1_ = 0.377, *p* = 0.003; r_2_ = 0.367, *p* = 0.004; r_3_ = 0.591, *p* < 0.001; r_4_ = 0.432, *p* < 0.001; r_5_ = 0.344, *p* = 0.007; r_6_ = −0.317, *p* = 0.014).

### 3.4 Predicative ability of uPAR for myocardial dysfunction

**Figure 5:**
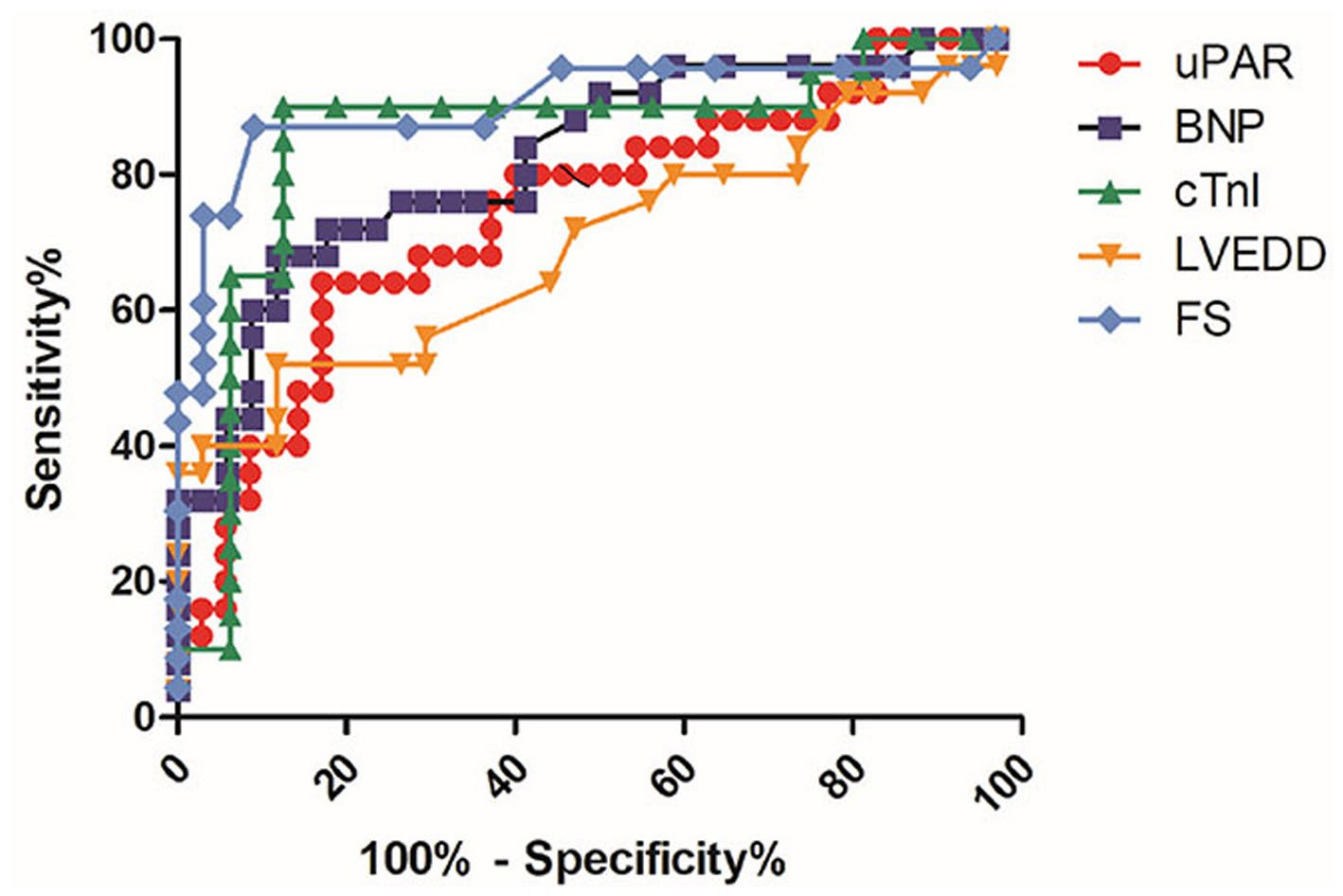
ROC curves. UPAR and some clinic parameters which were also differential between sepsis and SIC groups were used to compare their predictive values for SIC by ROC curve analysis (SIC, *n* = 25; sepsis, *n* = 25).

Receiver operating characteristic (ROC) curves and logistic regression analyses were used to evaluate the accuracy of uPAR in predicting myocardial dysfunction. ROC curve analysis showed that the area under curve (AUC) of uPAR was lower than that of BNP, cTnI, and FS, but higher than LVEDD (Table 3 and Figure 5), suggesting that the predictive value of uPAR was inferior to that of BNP, cTnI, and FS, while it was superior to LVEDD. Moreover, logistic regression model showed that uPAR was an independent predictor of myocardial dysfunction (*p* < 0.05).

**Table 3:** The results of ROC analysis.

Variable	AUC	Sensitivity (%)	Specificity (%)	95% confidence interval	*p-*value
uPAR	0.743	64.00	82.86	0.614–0.872	0.001
BNP	0.825	68.00	88.24	0.716–0.933	<0.001
cTnI	0.856	90.00	87.50	0.715–0.997	<0.001
LVEDD	0.698	52.00	88.24	0.556–0.841	0.01
FS	0.905	86.96	90.91	0.811–0.999	<0.001

## 4 Discussion

SIC is a serious complication of sepsis. Its prevalence in septic patients ranges between 10 and 70%, and it is associated with significantly increased rate of mortality ranging between 20 and 50% (11). However, the critical care community has not given significant emphasis to this syndrome, and formal diagnostic criteria are lacking. A proper diagnosis is needed to reduce mortality.

Circulating myocardium-depressing factors, such as TNF-α, cTnT, cTnI, and BNP, are the initial stimuli and driving forces of septic myocardial dysfunction. Although these cardiac biomarkers are clear indicators of the severity of illness and prognosis in patients with sepsis, none of them are specific to SIC. Sepsis is an inappropriate immune response that stimulates the expression of inflammatory cytokines to damage the myocardium. Circulating cytokines can activate cardiac fibroblasts and cardiomyocytes, which further stimulate iNOS expression and cause myocardial depression in sepsis. In addition, serum or plasma detection are important means for clinical diagnosis due to its ease of access, which makes clinical diagnosis of diseases simpler and quicker. Therefore, measuring serum cytokine levels may aid in early diagnosis of SIC.

In this study, we utilized a human antibody array to detect serum levels of 40 immune response-associated cytokines in sepsis patients with myocardial dysfunction, sepsis patients, and healthy individuals. Statistical analysis of the results revealed that resistin, thrombomodulin, uPAR, and CD163 were differential between SIC and sepsis patients; lipocalin-2, CRP, CD14, IL-13, uPAR, and ICAM-1 were differential between SIC patients and healthy population; while lipocalin-2, CRP, CD14, IL-13, PF4, FAS L, and ICAM-1 were differential between sepsis patients and healthy population. Venn diagram analysis showed that uPAR was different in the SIC group compared to the sepsis and healthy groups, but did not differ between the sepsis and healthy groups; planned contrast analyses further confirmed that uPAR elevation was disproportionately greater in SIC than in sepsis alone (*p* = 0.007), supporting uPAR may serve as a candidate biomarker associated with SIC. More importantly, uPAR levels significantly positively correlated with APACHE II, SOFA, BNP, and FS, indicating that uPAR may be involved in sepsis-associated myocardial dysfunction. Additionally, uPAR levels also significantly positively correlated with the use and duration of vasopressor therapy. While this observational association does not establish a causal or guiding role for uPAR in vasopressor management, it suggests that higher uPAR levels may reflect a more severe hemodynamic and inflammatory status requiring greater vasopressor support. Whether uPAR levels could contribute to clinical decision-making regarding vasopressor therapy warrants investigation in future interventional studies. APACHE II and SOFA scores proved to be the best indicators of sepsis. However, previous studies have reported that the predictive power of APACHE II in combination with IL-6 was higher than the predictive value of APACHE II alone (12), and a combination of SOFA score with NGAL provided better prediction of septic AKI and in-hospital mortality in critically ill surgical patients (13). Thus, the combination of these indicators and uPAR may improve prediction and diagnosis of SIC.

Vasopressors are useful in augmenting arterial blood pressure and cardiac function, and their use is included in the standard management of sepsis and sepsis-induced organ dysfunction. A previous study indicated that dobutamine doses were positively associated with TNF-α plasma levels, and dopamine doses were positively associated with IL-6, independent of disease severity, hemodynamics, and outcome in septic shock patients (14). Vasopressors have been suggested to modulate innate immunity (15). These findings, together with the observed correlation between uPAR and vasopressor use in our study, suggest that higher circulating uPAR levels in patients with SIC may contribute to a more severe immune response, which would require a higher vasopressor dose. In addition, ROC analysis and logistic regression model indicated that uPAR could be used as an independent predictor of myocardial dysfunction.

uPAR is a novel inflammatory marker released from neutrophils, lymphocytes, monocytes/macrophages, endothelial and tumor cells. It is involved in various cellular functions, including cell adhesion, migration, chemotaxis, and signal transduction. Numerous studies have shown increased systemic levels of uPAR in various infectious and inflammatory diseases, including immunodeficiency virus (HIV) infections (16), malaria (17), tuberculosis, central nervous system infections (18), arthritis (19), inflammatory bowel disease and sepsis (20). Importantly, although suPAR is increasingly being recognized as a potentially valuable biomarker for sepsis diagnosis and prognosis (21), its specific role in SIC has not been previously investigated. However, ours is the first study to have reported increased levels of uPAR in SIC. suPAR has been established as a biomarker of systemic chronic inflammation and immune activation across a broad spectrum of diseases (22, 23). In cardiovascular research, suPAR has been shown to be associated with early subclinical myocardial impairment even in patients with preserved ejection fraction (24), and is strongly predictive of adverse outcomes in heart failure, with its combination with BNP significantly improving risk prediction (25). Elevated plasma uPAR levels have further been associated with the incidence of heart failure (26) and independently predicted mortality in chronic heart failure patients (27). Therefore, it is hypothesized that elevated uPAR levels may induce heart failure via inflammatory mechanisms.

In addition, uPAR has been reported to be associated with various pro-inflammatory cytokines (28). For example, the uPAR/uPA system is regulated by deactivating cytokines (IL-4, IL-10, and IL-13), which result in adhesion of cells to vitronectin and fibrinogen (29). The uPA/uPAR system has been found to be active in immature dendritic cells derived from CD14^+^CD34^+^ precursors (30). In this study, we found that CD14 and IL-13 were increased in sepsis and SIC patients compared to the healthy population, suggesting that upregulated CD14 and IL-13 in sepsis might activate uPAR to induce myocardial dysfunction. Additionally, lipocalin-2, CRP and ICAM-1 levels were also elevated in sepsis and SIC compared to the healthy population. However, there have been no reports on the association of uPAR with lipocalin-2, CRP and ICAM-1. Notably, among the differentially expressed proteins between SIC and sepsis patients, resistin and CD163 were also significantly elevated in the SIC group. Both molecules are related to macrophage biology. Resistin has been proposed as a potential diagnostic biomarker for sepsis (31) and has also been associated with sepsis severity and mortality in critically ill septic patients (32). CD163 is a macrophage scavenger receptor involved in inflammatory and hemoglobin-scavenging pathways (33), and CD163-positive cardiac-resident macrophages have been shown to help maintain cardiomyocyte homeostasis during septic stress (34). The concurrent elevation of uPAR, resistin, and CD163 in SIC patients may therefore reflect a converging macrophage-associated inflammatory response contributing to myocardial injury, although the precise interactions among these biomarkers and their incremental diagnostic value for SIC require further investigation. CD14 (a glycoprotein expressed on the surface of monocytes/macrophages), IL-13 (an immunoregulatory and effector cytokine), lipocalin-2 (an innate immune protein), C-reactive protein (an acute-phase plasma protein for activation of immune system), and ICAM-1 (a key mediator for the interaction of immune cells) participate in the regulation of immune system, indicating that uPAR may induce myocardial dysfunction through inflammatory mechanisms. Furthermore, these cytokines have also been reported to be increased in sepsis in previous studies (35–38), suggesting that these cytokines may be potential biomarkers of sepsis.

Several limitations of this study warrant acknowledgment. First, the small sample size (*n* = 60) and single-center design limit the generalizability of our findings; the limited sample size also precluded robust subgroup analyses and may have increased the risk of overfitting in multivariable models. Second, suPAR was measured at a single time point within 24 h of enrollment, precluding assessment of its dynamic changes during sepsis progression and their relationship with the evolution of SIC; moreover, future studies should include both technical and biological replicates to further strengthen the robustness of biomarker evaluation. Third, as renal function is a known determinant of circulating suPAR levels, and given that some patients received CRRT, residual renal-related confounding cannot be entirely excluded (39). These limitations underscore the need for future multicenter validation studies incorporating non-septic critically ill controls, serial biomarker measurements, and independent ELISA-based confirmation to comprehensively establish the diagnostic and prognostic utility of suPAR in SIC.

In conclusion, our study was the first to find an association between uPAR and SIC. uPAR may induce myocardial dysfunction in septic patients via inflammatory mechanisms. Moreover, its specific elevation in SIC, along with its correlations with disease severity and cardiac parameters, supports its potential as a candidate biomarker. However, given the observational nature of this study, future *in vitro* and *in vivo* experiments are needed to validate these findings and elucidate the underlying pathophysiological mechanisms.

## 5 Conclusion

uPAR may serve as a candidate biomarker associated with SIC and may have potential value in guiding vasopressor use.

## Data Availability

The raw data supporting the conclusions of this article will be made available by the authors, without undue reservation.

## Footnotes

1
http://bioinformatics.psb.ugent.be/webtools/Venn/
